# Impact of Chronic Kidney Disease on Aortic Dissection in Patients with Polycystic Kidney Disease: A Fifteen-year Nationwide Population-based Cohort Study in Taiwan

**DOI:** 10.7150/ijms.106518

**Published:** 2025-02-26

**Authors:** Chia-Ning Fan, Chi-Hsiang Chung, Wu-Chien Chien, Chang-Huei Tsao, Tzu-Hsuan Weng, Kun-Lin Wu, Wen-Fang Chiang, Chih-Chien Yen, Jenq-Shyong Chan, Po-Jen Hsiao

**Affiliations:** 1 Department of Surgery, Taoyuan Armed Forces General Hospital, Taoyuan, Taiwan.; 2 Department of Surgery, National Defense Medical Center, Division of Cardiovascular Surgery, Tri-Service General Hospital, Taipei, Taiwan.; 3 Department of Medical Research, Tri-Service General Hospital, National Defense Medical Center, Taipei, Taiwan.; 4 School of Public Health, National Defense Medical Center, Taipei, Taiwan.; 5 Department of Microbiology & Immunology, National Defense Medical Center, Taipei, Taiwan.; 6 Division of Nephrology, Department of Internal Medicine, Tri-Service General Hospital, National Defense Medical Center, Taipei, Taiwan.; 7 Division of Nephrology, Department of Internal Medicine, Taoyuan Armed Forces General Hospital, Taoyuan City, Taiwan.; 8 Department of Biomedical Sciences and Engineering, Institute of Systems Biology and Bioinformatics, National Central University, Taoyuan, Taiwan.; 9 Department of Life Sciences, National Central University, Taoyuan City, Taiwan.

**Keywords:** polycystic kidney disease, aortic dissection, chronic kidney disease, end-stage renal disease, hemodialysis, cardiovascular risk

## Abstract

**Background:** Aortic dissection is a life-threatening condition associated with polycystic kidney disease (PKD). Additionally, PKD often progresses to chronic kidney disease (CKD), a known risk factor for cardiovascular disease. However, the impact of CKD on aortic dissection, particularly in patients with PKD, remains unclear. This study aims to investigate the effects of both CKD and PKD on aortic dissection.

**Materials and methods:** This nationwide, population-based, retrospective cohort study used data from the National Health Insurance Research Database (NHIRD) in Taiwan. The primary outcome evaluated in this study was the cumulative incidence of aortic dissection, compared between PKD patients and a control group without PKD over a 15-year follow-up period. CKD subgroup analyses were performed to further assess the impact of CKD progression on the development of aortic dissection.

**Results:** From 2000 to 2015, this study included 9,192 PKD patients and 36,768 matched controls without PKD from the NHIRD. Our findings demonstrated that PKD patients who developed aortic dissection had a higher incidence of comorbidities, including hypertension and coronary artery disease. Aortic dissection was more prevalent among male patients, individuals over 45 years of age, and those in the lowest insured premium group. PKD patients had a 2.53-fold higher adjusted hazard ratio (HR) for developing aortic dissection compared to the control group (95% CI: 1.74 to 3.66, p < 0.001). Notably, PKD patients with concurrent hypertension had a 7.77-fold increased risk of aortic dissection (95% CI: 4.97 to 12.13, p < 0.001). In CKD subgroup analyses, PKD patients without CKD and those with CKD had adjusted HRs of 1.74 and 3.38, respectively (p < 0.001). Among PKD patients with CKD, those who initiated hemodialysis (HD) and those who did not showed adjusted HRs of 3.95 and 2.74, respectively, for aortic dissection (p < 0.001).

**Conclusion:** These findings indicate that the risk of aortic dissection in PKD patients significantly increases with CKD progression. Additionally, hypertension is an independent risk factor for aortic dissection in PKD patients. Careful management of blood pressure and strategies to prevent CKD progression may reduce the incidence of aortic dissection in this population.

## Introduction

Acute aortic dissection is a life-threatening condition within the spectrum of acute aortic syndromes and has often been misdiagnosed and underestimated in past decades 1, 2. The incidence of aortic dissection is estimated to be 3-30 cases per million people per year, with most data derived from autopsy studies. Identifying and assessing the risk factors contributing to aortic dissection is essential. Previous population-based cohort studies have provided data on the incidence and traditional risk factors for aortic dissection, including cardiovascular diseases, uncontrolled hypertension, and atherosclerosis 3-5.

PKD, the most common hereditary kidney disease, affecting an estimated 1 in 1,000 to 1 in 2,500 individuals worldwide. PKD leads to a progressive decline in renal function, often culminating in end-stage renal disease, and is a multisystem disorder with numerous clinical manifestations, which may result in early mortality for some patients 6, 7. Major cardiovascular complications reported in PKD patients include acute coronary syndrome, stroke, and congestive heart failure 8, 9. Aortic dissection, though potentially catastrophic in PKD patients, has only been reported in a few studies 10, 11. Unlike cerebral aneurysms, aortic dissections are more challenging to screen for before severe complication arise, except in cases of pre-existing aneurysmal change. Current literature suggests an association between PKD and aortic dissection 11, 12. CKD itself also increases the risk of AD-related mortality, suggesting that its development may be a target for prevention and early identification of high-risk individuals in the general population 13. However, the mechanism linking CKD to aortic dissection remains unclear. This study investigates the association between aortic dissection, PKD, and CKD in PKD patients, drawing on data from Taiwan's NHIRD, which includes records for the majority of Taiwan's residents and is widely used for epidemiological research.

## Materials and methods

### Data source

The NHIRD contains outpatient and inpatient claims for all enrollees in Taiwan's mandatory National Health Insurance (NHI) program, representing more than 99% of the Taiwanese population (over 23 million people). The NHIRD includes patient identifiers, birthdates, sex, dates of admission and discharge, ICD-9-CM (International Classification of Disease, 9^th^ Revision, Clinical Modification) diagnostic codes (up to five per case), and outcomes. This study utilized data from the Longitudinal Health Insurance Database (LHID), a randomly selected subset of the NHIRD containing information on approximately one million beneficiaries, representing around 5% of Taiwan's population. Data were randomly selected from the NHIRD between 2000 and 2015. Previous studies have validated the accuracy of major diseases diagnoses in the NHIRD, including polycystic kidney disease (PKD) and aortic dissection 9, 14. This study was approved by the Institutional Review Board of Tri-Service General Hospital at the National Defense Medical Center in Taipei, Taiwan (TSGH IRB E202416015). The requirement for informed patient consent was waived.

### Sampled patients

In this Taiwan's NHI research, all diagnoses were defined using the ICD-9-CM. The study included a PKD cohort and a comparison cohort. Patients aged ≥20 years newly diagnosed with cystic kidney disease (ICD-9-CM 753.12-753.14: polycystic kidney, adult type, autosomal dominant, autosomal recessive) were followed from 2000 to 2015. Patients were excluded if they had a history of PKD before the inclusion date, were under 20 years of age, had unknown sex, lacked follow-up, or had an initial diagnosis of aortic dissection (ICD-9-CM 441.0). The PKD diagnosis date was set as the inclusion date. Control patients were randomly selected from individuals in the LHID based on age, sex, comorbidities and insured premium level, excluding those with a history of PKD. CKD is defined by ICD-9-CM diagnostic codes system including from ICD-9-CM codes 580 to 589 including glomerulonephritis, nephrotic syndrome and renal failure.

Pre-existing comorbidities were assessed for each participant, including diabetes mellitus (ICD-9-CM codes 250), hypertension (401-405), hyperlipidemia (272), coronary artery disease (410-414), stroke (430-438), congestive heart failure (428), peripheral arterial occlusive disease (443.9), atrial fibrillation (427.31), chronic obstructive pulmonary disease (490-496), chest trauma (338.11, 338.21, 862, 901.0, 959.1), and cancer (140-239). Insured premiums (in New Taiwan dollars, NTD) were stratified into three categories according to enrollee's monthly salary.

### Statistical analysis

This population-based, retrospective cohort study used SPSS software version 22 (SPSS Inc., Chicago, Illinois, USA) for all analyses. Standardized difference and standardized mean difference were presented for categorical and continuous variable distributions, respectively. In this study, the Kolmogorov-Smirnov test was used to assess whether the dataset followed a normal distribution. Chi-square (χ²) tests and t-tests were conducted, with t-tests used for continuous variables. Results are reported as hazard ratios (HR) with 95% confidence intervals (CI) and Wald test statistics. The AD-free survival rate between PKD and non-PKD group was analyzed using Kaplan-Meier method. Multivariable cox proportional hazards regression analysis determined the risk of aortic dissection in patients with and without PKD, clarifying the impact of each comorbidity. Subgroup analyses were performed to evaluate the risk of aortic dissection in PKD patients with and without CKD, as well as in those with end-stage renal disease (ESRD) undergoing hemodialysis.

## Results

**Figure 1:**
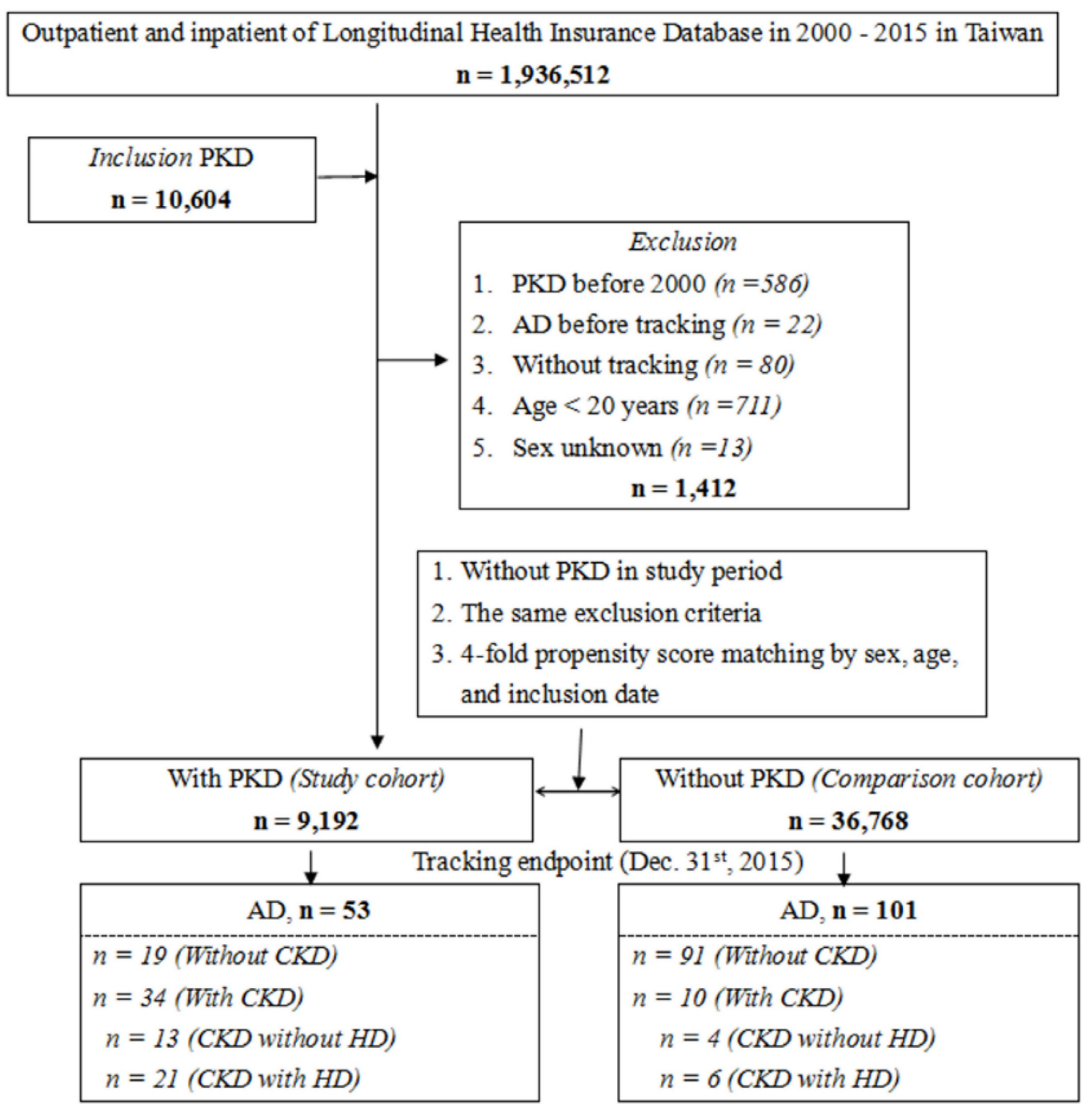
Flowchart of the study. Abbreviations: AD = aortic dissection, CKD = chronic kidney disease, HD = hemodialysis, PKD = polycystic kidney disease.

**Figure 2:**
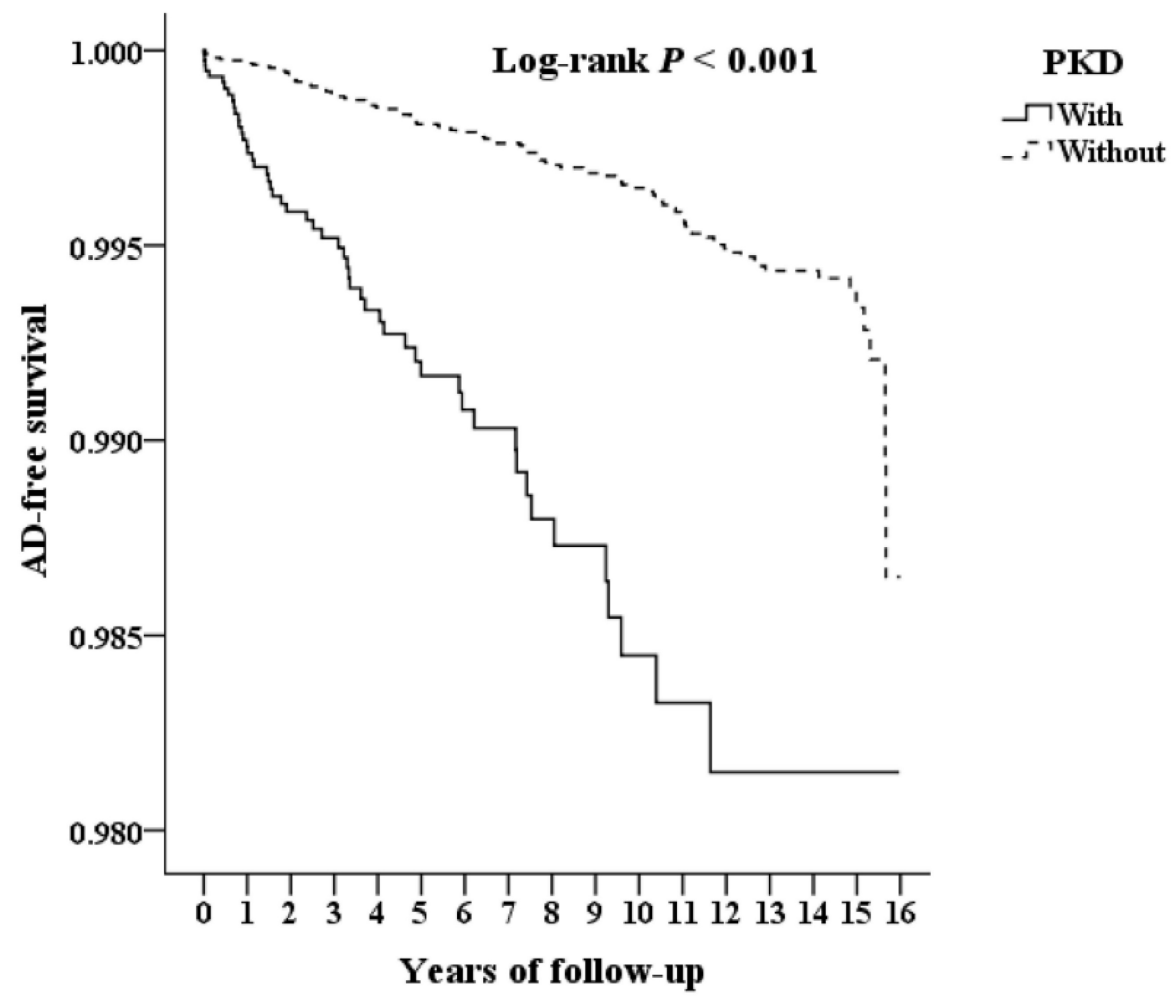
Kaplan-Meier for cumulative survival of AD aged 20 and over stratified by PKD with log-rank test. Abbreviations: AD = aortic dissection, PKD = polycystic kidney disease.

The flowchart of patient enrollment from 2000 to 2015 is shown in Figure 1. Among the 1,936,512 patients in the LHID from the NHIRD, 9,192 PKD patients were identified. The cumulative incidence of aortic dissection during the 15-year follow-up was recorded for PKD patients and the control group and further analyzed according to CKD status. Kaplan-Meier analysis (Figure 2) shows a significantly lower AD-free survival rate in PKD patients compared to the non-PKD group (p < 0.001) in the log-rank test over the 15-year follow-up. Baseline characteristics of the study subjects and controls, including sex, age, income, and comorbidities, are presented in Table 1. Compared with controls (non-PKD group), PKD patients had higher rate of hypertension (p < 0.001), coronary artery disease (p < 0.001), congestive heart failure (p < 0.001) and chronic kidney disease (p < 0.001); but lower rates of diabetes mellitus (p < 0.001), hyperlipidemia (p = 0.011), stroke (p < 0.001), chronic obstructive pulmonary disease (p < 0.001), and chest trauma (p = 0.048).

**Figure 3:**
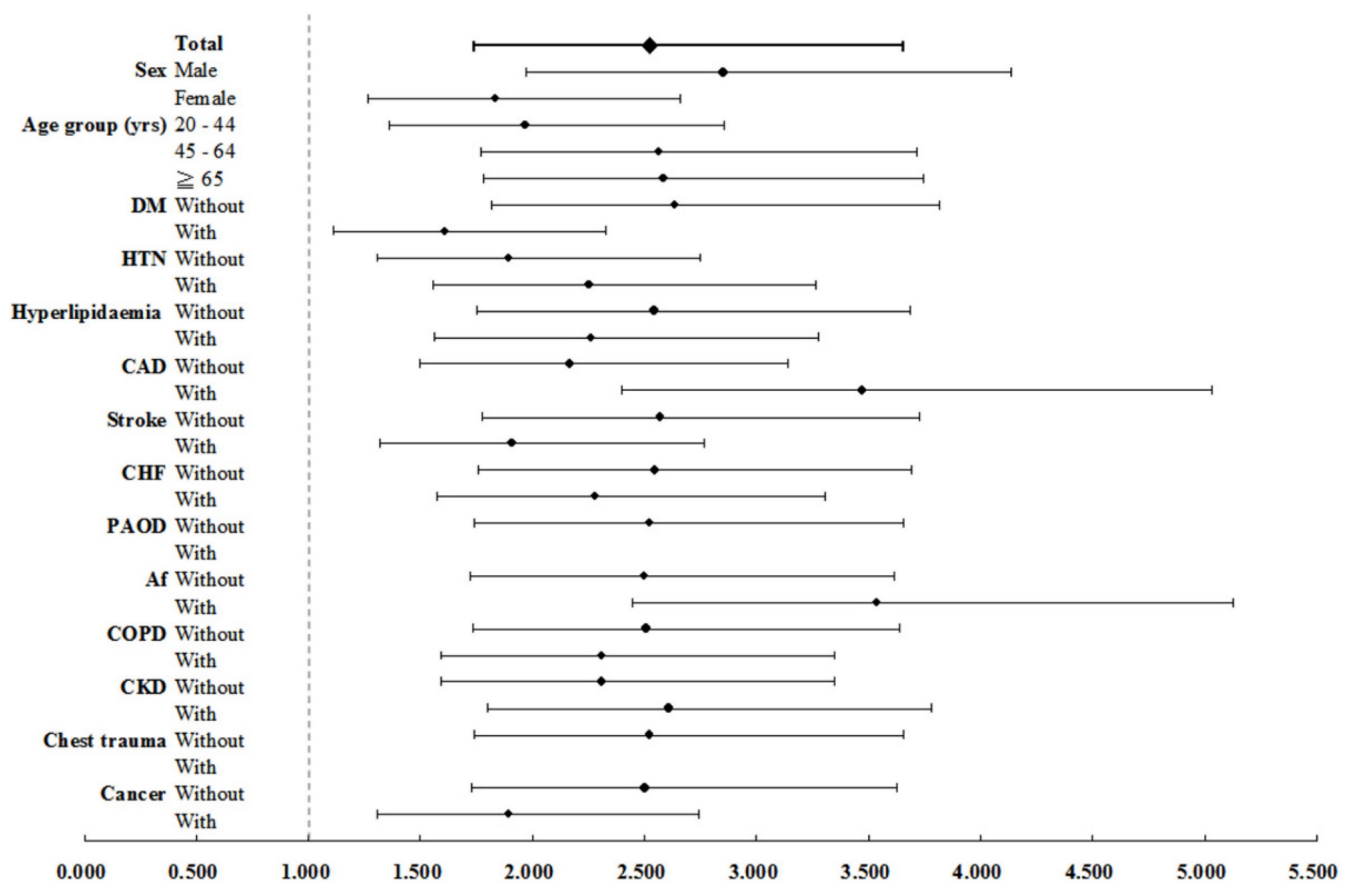
Forest plot for factors of AD stratified by sex, age groups, and comorbidities. Abbreviations: AD = aortic dissection, DM = diabetes mellitus, HTN = hypertension, CAD = coronary artery disease, CHF = congestive heart failure, PAOD = peripheral arterial occlusive disease, Af = atrial fibrillation, COPD = chronic obstructive pulmonary disease, CKD = chronic kidney disease.

In Table 2, the incidence rate ratio of aortic dissection in PKD versus non-PKD was 4.05 (95% CI: 2.87 to 5.71, p < 0.001). After adjusting for age, sex, and comorbidities using multivariate Cox regression, PKD patients had a 2.53-fold higher risk of developing aortic dissection than non-PKD patients (95% CI: 1.74 to 3.66, p < 0.001). Additionally, male sex and hypertension were identified as independent risk factors for aortic dissection (both p < 0.001), while cancer was associated with a lower risk (adjust HR 0.33, 95% CI: 0.15 to 0.71, p < 0.005). Table 3 shows the incidence rates of aortic dissection in PKD patients and non-PKD patients were 60.44 and 27.44 per 100000 person-years, respectively. PKD patients had a 2.53-fold increased risk of aortic dissection as compared with controls. Adjust HR for aortic dissection was significantly elevated across subgroups of both sexes, all ages, and patients with or without atherosclerotic risk factors or comorbidities, including hypertension, diabetes mellitus, hyperlipidemia, coronary artery disease, stroke, congestive heart failure, atrial fibrillation, chronic obstructive pulmonary disease and CKD (all p < 0.001) (Figure 3). Subgroup analysis (Table 4) examined the impact of CKD on aortic dissection. PKD patients without CKD and those with CKD had adjusted HRs of 1.74 and 3.38, respectively, after adjusting for age, sex, and comorbidities (p < 0.001). Among PKD patients with CKD on hemodialysis and those without hemodialysis showed adjusted HRs of 3.95 and 2.74, respectively (p < 0.001). In the non-PKD group, CKD patients on hemodialysis had an adjusted HR of 1.58 (p = 0.001).

In Table S1 and Figure S1, after adjusting for age, sex, and comorbidities, hypertension and PKD were associated with adjusted HRs of 2.83 and 2.18 for aortic dissection (p < 0.001 and p = 0.014, respectively). PKD patients with hypertension had a 7.77-fold increased risk of aortic dissection (p < 0.001). These results suggest that hypertension significantly increases the risk of aortic dissection in PKD patients.

## Discussion

This study is the first to investigate the relationship between CKD and aortic dissection in patients with PKD. In PKD patients, the risk of aortic dissection is notably increased by the presence of hypertension and the progressive decline in renal function, characteristic of CKD. We purpose this increased risk may result from vascular remodeling and heightened hemodynamic stress associated with PKD, which can weaken the aortic wall over time.

### Polycystic kidney disease and associated complications

PKD is a genetic disorder characterized by mutations in the PKD1 and PKD2 genes, which disrupt the integrity of polycystin1 and polycystin2 in vascular smooth muscle 15. In addition to impaired renal function, PKD patients frequently experience other systemic complications, including hypertension, liver or pancreatic cysts, intracranial aneurysms, diverticular disease, and cardiac valve abnormalities 16-18. Mortality in PKD patients frequently results from cardiac complications, infection and central nervous system disorders, often due to ruptured intracranial aneurysms 19. Clinical guidelines recommend antihypertensive treatment and intracranial aneurysm screening to improve the quality of life and prognosis for PKD patients 20. Our findings indicate that PKD patients have a higher incidence of comorbidities such as hypertension, coronary artery disease, congestive heart failure, and CKD. The incidence rate of aortic dissection in PKD patients is 2.52 times higher than in non-PKD patients. Vascular disease, such as aneurysms and arterial dissection of large arteries (e.g., the aorta and coronary arteries), are significant causes of mortality in PKD 12. However, the incidence of abdominal aortic aneurysms does not appear to be elevated in PKD patients 21, 22.

The PKD1 and PKD2 genes play complex roles in mechano-transduction and intracellular calcium signaling, which are critical in the molecular basis of intracerebral aneurysm formation 23, 24. Studies have shown that PKD patients exhibit endothelial dysfunction and hypertension, potentially due to reduced nitric oxide, oxidative stress, and antioxidant imbalance 25, 26. There is also evidence that PKD patients have a higher incidence of aortic dilatation, a known risk factor for aortic dissection 27. However, PKD patients with intracranial aneurysm do not always display the same tendency toward aortic and coronary aneurysms, aortic root dilatation, or aortic dissection, suggesting that the mechanism for intracranial aneurysm formation in PKD may differ from that of aortic aneurysm or dissection 28.

Hypertension is the most common risk factor for acute aortic dissection, contributing to 75% of cases. Other contributing factors include trauma, pharmacologic factors, vasculitis, infection, and genetic factors such as bicuspid aortic valve and connective tissue disorders 29. Pathologically, acute aortic syndrome can be divided into few conditions such as classic dissection, intramural hematoma, penetrating aortic ulcer, symptomatic aneurysm or pseudoaneurysm and aortic rupture 30, 31. Classic aortic dissection and intramural hematoma are more related to structural and genetic conditions, while penetrating aortic ulcer is more associated with atherosclerosis 32. These risk factors can be categorized into conditions that increase aortic wall stress, aortic media abnormalities, and iatrogenic factors. Genetic syndromes and inflammatory vasculitis contribute to aortic media abnormalities 31. Advances in molecular genetics and imaging have significantly enhanced the prediction and prevention of acute aortic dissection 3, 33-35. Genetically related aortic syndromes, typically autosomal dominant, often affect younger individuals and are linked to connective tissue disorders in 20% of cases. Mutations affecting the contractile apparatus of vascular smooth muscle cells suggest that smooth muscle tone and function are crucial for aortic wall stress response 29. Using the ClinGen framework, genes predisposing to heritable thoracic aortic aneurysms and dissection have been identified, though PKD1 and PKD2 mutations are considered risk alleles with limited evidence as a Mendelian cause of heritable thoracic aortic aneurysm and dissection 34. Therefore, PKD1 and PKD2 gene abnormalities alone may not fully explain the increased risk of aortic dissection in PKD, warranting further investigation.

Approximately 60% of PKD patients develop early-onset hypertension before renal function declines, typically around age 29 36, 37. While the optimal blood pressure target for PKD patients is not well established, it is generally considered similar to that for CKD patients without PKD. For most PKD patients, the goal blood pressure should be 120-125/< 80 mmHg, with angiotensin-converting enzyme inhibitors (ACEIs) as the preferred treatment and angiotensin II receptor blockers (ARBs) as an alternative. Lower blood pressure targets may slow kidney volume increase and provide cardiovascular benefits in younger, healthier PKD patients 38, 39. Literature indicates that PKD patients have a higher incidence of aortic dissection, with a younger average age and a higher prevalence of hypertension compared to non-PKD patients 11, 12. Among PKD patients with hypertension, our study found a significantly elevated incidence of aortic dissection, with a 7.91-fold increase in risk, suggesting that hypertension significantly exacerbates the risk of aortic dissection in PKD patients. A previous NHIRD study with 2,076 PKD patients followed for 12 years identified hypertension as an aggravating factor in developing aortic dissection 14. However, the role of CKD itself in developing aortic dissection in PKD patients was not identified in this study. CKD is a known risk factor for aortic dissection-related mortality in the general population 13. Nevertheless, its impact on aortic dissection in PKD patients has not been widely studied in large datasets. In our study, Cox regression analysis of PKD-associated aortic dissection showed an elevated risk in patients with CKD, with adjusted hazard ratios of 3.38 (p < 0.001). The risk was even higher in PKD patients requiring hemodialysis, with an adjusted hazard ratio of 3.95 (p < 0.001). These findings suggest that aortic dissection risk in PKD patients rises with CKD.

### Therapeutic strategies to slow CKD progression in PKD patients

PKD is a genetic disorder characterized by the formation of cysts in the kidneys. These cysts cause the kidneys to enlarge and may lead to damage. Kidney cyst number increment and volume increase are indicators of PKD progression. The decline in kidney function in patients with PKD typically occurs at variable rates, usually depending on age and the kidney cystic burden or number of cysts. Rapid progression refers to these patients who reach kidney failure at an earlier age 40, 41. The progressive enlargement of multiple cysts results in the deterioration of functional parenchyma, potentially leading to end-stage kidney disease. There are two main types of PKD: autosomal dominant polycystic kidney disease (ADPKD) and autosomal recessive polycystic kidney disease (ARPKD). ADPKD is far more common than ARPKD in clinical practice. Both ADPKD and ARPKD results from abnormal genes that can be inherited from a parent or, in rare cases, arise due to spontaneous genetic mutations in individuals with no family history of the disease 40-42. COX inhibition with Sulindac significantly reduced kidney size of the PKD2 mice, suggesting the COX pathway may be a therapeutic target for PKD. However, the long-term efficacy and side effects of non-steroidal anti-inflammatory drugs (NSAIDs) in managing cystic renal disease remain uncertain 40. Tolvaptan, a vasopressin V2 receptor antagonist, is an approved treatment for PKD that helps to reduce kidney volume and slow renal function deterioration 41, 42. Additionally, Tolvaptan may hold potential for treating aortic aneurysms and dissection by preserving aortic integrity, reducing inflammatory markers, and inhibiting vascular smooth muscle cell apoptosis 43. Combined sodium-glucose cotransporter-2 (SGLT2) inhibitors and ACEIs/ARBs, known for their glycemic and cardiorenal benefits, showed therapeutic effects in patients with CKD 44. Inducing glucosuria with the SGLT2-specific inhibitor dapagliflozin has been associated with improved renal function and reduced albuminuria in PKD rat models 45. However, in examined patients, short-term dapagliflozin administration led to a decrease in eGFR and an increase in height-adjusted kidney volume (htTKV) 46. SGLT2 inhibitors may offer additional benefits 47-49; however, data on their safety and effects in patients with ADPKD are currently lacking, as these patients were excluded from SGLT2 inhibitors trials. Notably, there is speculation that SGLT2 inhibitors could promote cysts growth and accelerate kidney function decline in ADPKD 46, 50, 51. Now, the ongoing EMPA-PKD trial is evaluating the safety of empagliflozin in patients with rapidly progressive ADPKD, both with and without concurrent tolvaptan use, by monitoring total kidney volume and kidney function decline 52. Further studies, including clinical trials, are essential to evaluate the efficacy of SGLT2 inhibitors in patients with PKD. In summary, routine health management, especially blood pressure control and CKD prevention or early detection, may help reduce the likelihood of aortic dissection in PKD patients.

### Limitation

Despite extensive adjustments through matching and multivariate logistic regression, our study has several limitations. The NHIRD database presents specific challenges: 1) as a study of a Taiwanese population, its generalizability to other countries is limited; 2) potential misclassification of disease diagnoses, as diagnosis codes may overestimate or underestimate actual conditions to qualify for NHIRD benefits; 3) limitations in result extrapolation due to the specific nature of NHIRD, its unique healthcare ecosystem, and the parent group's large sample size, which restricts over-interpretation based on statistically significant differences; and 4) the choice of control group could influence result interpretation.

## Conclusion

The risk of aortic dissection in patients with PKD significantly increases with hypertension and CKD. Effective clinical management should focus on achieving optimal blood pressure control, utilizing antihypertensive agents such as ACEIs/ARBs, and employing renoprotective strategies among these patients. Regular cardiovascular and renal function monitoring is critical to mitigating the risk of life-threatening aortic events and managing PKD-related cardiovascular complications.

## Supplementary Material

Supplementary figure.

## Figures and Tables

**Table 1:** Baseline characteristics of patients with aortic dissection

PKD	Total		With		Without		*P*
Variables	n	%	n	%	n	%	
Total	45,960		9,192	20.00	36,768	80.00	
Sex							0.999
Male	26,035	56.65	5,207	56.65	20,828	56.65	
Female	19,925	43.35	3,985	43.35	15,940	43.35	
Age (years)	56.65 ± 16.13		56.79 ± 15.39		56.62 ± 16.31		0.366
Age groups (years)							0.999
20 - 44	10,055	21.88	2,011	21.88	8,044	21.88	
45 - 64	19,435	42.29	3,887	42.29	15,548	42.29	
≧ 65	16,470	35.84	3,294	35.84	13,176	35.84	
Insured premium (NT$)							0.083
< 18,000	45,268	98.49	9,031	98.25	36,237	98.56	
18,000 - 34,999	522	1.14	119	1.29	403	1.10	
≧ 35,000	170	0.37	42	0.46	128	0.35	
DM							< 0.001
Without	40,568	88.27	8,383	91.20	32,185	87.54	
With	5,392	11.73	809	8.80	4,583	12.46	
HTN							< 0.001
Without	36,993	80.49	5,708	62.10	31,285	85.09	
With	8,967	19.51	3,484	37.90	5,483	14.91	
Hyperlipidemia							0.011
Without	44,727	97.32	8,981	97.70	35,746	97.22	
With	1,233	2.68	211	2.30	1,022	2.78	
CAD							< 0.001
Without	42,217	91.86	8,350	90.84	33,867	92.11	
With	3,743	8.14	842	9.16	2,901	7.89	
Stroke							< 0.001
Without	42,679	92.86	8,652	94.13	34,027	92.55	
With	3,281	7.14	540	5.87	2,741	7.45	
CHF							< 0.001
Without	44,897	97.69	8,927	97.12	35,970	97.83	
With	1,063	2.31	265	2.88	798	2.17	
PAOD							0.224
Without	45,943	99.96	9,191	99.99	36,752	99.96	
With	17	0.04	1	0.01	16	0.04	
Af							0.403
Without	45,451	98.89	9,098	98.98	36,353	98.87	
With	509	1.11	94	1.02	415	1.13	
COPD							< 0.001
Without	42,796	93.12	8,781	95.53	34,015	92.51	
With	3,164	6.88	411	4.47	2,753	7.49	
CKD							< 0.001
Without	42,050	91.49	6,721	73.12	35,329	96.09	
With	3,910	8.51	2,471	26.88	1,439	3.91	
Chest trauma							0.048
Without	45,927	99.93	9,190	99.98	36,737	99.92	
With	33	0.07	2	0.02	31	0.08	
Cancer							0.852
Without	42,833	93.20	8,571	93.24	34,262	93.18	
With	3,127	6.80	621	6.76	2,506	6.82	
CCI_R	0.14 ± 0.40		0.16 ± 0.45		0.13 ± 0.39		< 0.001
Season							0.999
Spring (Mar - May)	12,375	26.93	2,475	26.93	9,900	26.93	
Summer (Jun - Aug)	11,425	24.86	2,285	24.86	9,140	24.86	
Autumn (Sep - Nov)	11,215	24.40	2,243	24.40	8,972	24.40	
Winter (Dec - Feb)	10,945	23.81	2,189	23.81	8,756	23.81	
Location							< 0.001
Northern Taiwan	18,599	40.47	4,145	45.09	14,454	39.31	
Middle Taiwan	12,555	27.32	2,242	24.39	10,313	28.05	
Southern Taiwan	11,787	25.65	2,268	24.67	9,519	25.89	
Eastern Taiwan	2,805	6.10	509	5.54	2,296	6.24	
Outlets islands	214	0.47	28	0.30	186	0.51	
Urbanization level							< 0.001
1 (The highest)	15,878	34.55	3,563	38.76	12,315	33.49	
2	19,675	42.81	4,042	43.97	15,633	42.52	
3	3,221	7.01	477	5.19	2,744	7.46	
4 (The lowest)	7,186	15.64	1,110	12.08	6,076	16.53	
Level of care							< 0.001
Hospital center	15,539	33.81	4,120	44.82	11,419	31.06	
Regional hospital	14,845	32.30	3,662	39.84	11,183	30.42	
Local hospital	15,576	33.89	1,410	15.34	14,166	38.53	

*P:* Chi-square / Fisher exact test on category variables and t-test on continue variables Abbreviations: PKD = polycystic kidney disease, NT$ = new Taiwan dollar, DM = diabetes mellitus, HTN = hypertension, CAD = coronary artery disease, CHF = congestive heart failure, PAOD = peripheral arterial occlusive disease, Af = atrial fibrillation, COPD = chronic obstructive pulmonary disease, CKD = chronic kidney disease

**Table 2:** Factors of aortic dissection by using Cox regression

Variables	Crude HR	95% CI	95% CI	*P*	aHR	95% CI	95% CI	*P*
PKD								
Without	1.000				1.000			
With	4.054	2.877	5.713	< 0.001	2.525	1.742	3.658	< 0.001
Sex								
Male	2.063	1.451	2.934	< 0.001	2.178	1.528	3.104	< 0.001
Female	1.000				1.000			
Age groups (years)								
20 - 44	1.000				1.000			
45 - 64	2.669	1.212	5.879	0.015	1.786	0.803	3.972	0.155
≥ 65	2.279	1.056	4.916	0.036	1.805	0.821	3.968	0.142
Insured premium (NT$)								
< 18,000	1.000				1.000			
18,000 - 34,999	0.484	0.068	3.457	0.469	0.494	0.069	3.530	0.482
≥ 35,000	0.000	-	-	0.943	0.000	-	-	0.939
DM								
Without	1.000				1.000			
With	0.755	0.492	1.160	0.200	0.660	0.425	1.025	0.064
HTN								
Without	1.000				1.000			
With	3.640	2.635	5.030	< 0.001	3.102	2.182	4.409	< 0.001
Hyperlipidaemia								
Without	1.000				1.000			
With	1.173	0.518	2.655	0.702	0.751	0.324	1.742	0.505
CAD								
Without	1.000				1.000			
With	1.790	1.189	2.697	0.005	1.204	0.784	1.848	0.396
Stroke								
Without	1.000				1.000			
With	0.883	0.500	1.558	0.666	0.715	0.402	1.272	0.254
CHF								
Without	1.000				1.000			
With	1.107	0.583	2.101	0.756	0.955	0.493	1.853	0.893
PAOD								
Without	1.000				1.000			
With	0.000	-	-	0.857	0.000	-	-	0.976
Af								
Without	1.000				1.000			
With	1.276	0.523	3.110	0.593	1.086	0.437	2.699	0.860
COPD								
Without	1.000				1.000			
With	0.686	0.361	1.303	0.249	0.793	0.412	1.526	0.487
CKD								
Without	1.000				1.000			
With	1.563	1.010	2.419	0.045	1.423	0.894	2.266	0.137
Chest trauma								
Without	1.000				1.000			
With	0.000	-	-	0.879	0.000	-	-	0.988
Cancer								
Without	1.000				1.000			
With	0.313	0.147	0.668	0.003	0.311	0.144	0.671	0.003
CCI_R	1.044	0.557	1.278	0.423	1.042	0.623	1.425	0.778
Season								
Spring	1.000				1.000			
Summer	1.196	0.762	1.875	0.436	1.186	0.756	1.862	0.457
Autumn	0.827	0.514	1.329	0.432	0.806	0.501	1.297	0.375
Winter	1.181	0.750	1.859	0.472	1.172	0.744	1.846	0.493
Location					Multicollinearity with urbanization level			
Northern Taiwan	1.000				Multicollinearity with urbanization level			
Middle Taiwan	0.894	0.606	1.319	0.572	Multicollinearity with urbanization level			
Southern Taiwan	0.931	0.625	1.386	0.724	Multicollinearity with urbanization level			
Eastern Taiwan	0.784	0.390	1.576	0.495	Multicollinearity with urbanization level			
Outlets islands	0.000	-	-	0.935	Multicollinearity with urbanization level			
Urbanization level								
1 (The highest)	3.592	1.843	7.000	< 0.001	1.952	0.947	4.024	0.070
2	2.738	1.415	5.300	0.003	1.725	0.863	3.447	0.123
3	1.591	0.606	4.179	0.346	1.541	0.586	4.050	0.381
4 (The lowest)	1.000				1.000			
Level of care								
Hospital center	4.368	2.439	7.824	< 0.001	2.799	1.489	5.262	0.001
Regional hospital	2.050	1.119	3.757	0.020	1.641	0.888	3.033	0.114
Local hospital	1.000				1.000			

aHR = Adjusted hazard ratio: Adjusted variables listed in the table, CI = confidence interval Abbreviations: PKD = polycystic kidney disease, NT$ = new Taiwan dollar, DM = diabetes mellitus, HTN = hypertension, CAD = coronary artery disease, CHF = congestive heart failure, PAOD = peripheral arterial occlusive disease, Af = atrial fibrillation, COPD = chronic obstructive pulmonary disease, CKD = chronic kidney disease

**Table 3:** Factors of aortic dissection by using Cox regression

PKD	With			Without *(Reference)*			With vs. Without* (Reference)*			
Stratified	Events	PYs	Rate	Events	PYs	Rate	aHR	95% CI	95% CI	*P*
Total	53	87,686.41	60.44	101	368,082.64	27.44	2.525	1.742	3.658	< 0.001
Sex										
Male	41	48,152.67	85.15	70	204,665.22	34.20	2.853	1.969	4.135	< 0.001
Female	12	39,533.74	30.35	31	163,417.43	18.97	1.834	1.266	2.657	< 0.001
Age groups (yrs)										
20 - 44	5	9,034.30	55.34	12	37,246.23	32.22	1.969	1.359	2.853	< 0.001
45 - 64	22	34,961.79	62.93	28	99,577.15	28.12	2.565	1.770	3.717	< 0.001
≥ 65	26	43,690.33	59.51	61	231,259.26	26.38	2.586	1.784	3.747	< 0.001
Insured premium (NT$)										
< 18,000	52	86,180.19	60.34	101	361,883.48	27.91	2.478	1.710	3.591	< 0.001
18,000 - 34,999	1	1,139.05	87.79	0	5,014.88	0.00	∞	-	-	0.999
≥ 35,000	0	367.16	0.00	0	1,184.28	0.00	-	-	-	-
DM										
Without	49	76,928.06	63.70	80	288,824.80	27.70	2.636	1.819	3.819	< 0.001
With	4	10,758.35	37.18	21	79,257.84	26.50	1.608	1.110	2.331	< 0.001
HTN										
Without	15	51,472.01	29.14	47	266,892.02	17.61	1.897	1.309	2.748	< 0.001
With	38	36,214.40	104.93	54	101,190.62	53.36	2.254	1.555	3.266	< 0.001
Hyperlipidemia										
Without	51	85,163.30	59.88	96	355,641.00	26.99	2.543	1.755	3.685	< 0.001
With	2	2,523.11	79.27	5	12,441.64	40.19	2.261	1.560	3.276	< 0.001
CAD										
Without	35	76,901.21	45.51	79	328,144.93	24.07	2.167	1.495	3.140	< 0.001
With	18	10,785.20	166.90	22	39,937.71	55.09	3.472	2.396	5.032	< 0.001
Stroke										
Without	50	80,573.71	62.05	92	332,545.44	27.67	2.571	1.774	3.725	< 0.001
With	3	7,112.70	42.18	9	35,537.20	25.33	1.909	1.317	2.766	< 0.001
CHF										
Without	48	82,263.05	58.35	91	346,494.67	26.26	2.546	1.757	3.690	< 0.001
With	5	5,423.36	92.19	10	21,587.97	46.32	2.281	1.574	3.305	< 0.001
PAOD										
Without	53	87,640.68	60.47	101	367,925.17	27.45	2.525	1.742	3.658	< 0.001
With	0	45.73	0.00	0	157.47	0.00	-	-	-	-
Af										
Without	51	85,621.47	59.56	98	358,518.06	27.33	2.497	1.723	3.619	< 0.001
With	2	2,064.94	96.85	3	9,564.58	31.37	3.539	2.442	5.128	< 0.001
COPD										
Without	51	83,268.60	61.25	93	332,486.75	27.97	2.510	1.732	3.637	< 0.001
With	2	4,417.81	45.27	8	35,595.89	22.47	2.309	1.593	3.345	< 0.001
CKD										
Without	34	62,642.24	54.28	91	338,065.87	26.92	2.311	1.595	3.349	< 0.001
With	19	25,044.17	75.87	10	30,016.77	33.31	2.610	1.801	3.782	< 0.001
Chest trauma										
Without	53	87,686.41	60.44	101	367,959.13	27.45	2.525	1.742	3.658	< 0.001
With	0	0.00	-	0	123.51	0.00	-	-	-	-
Cancer										
Without	51	78,996.95	64.56	94	317,845.17	29.57	2.502	1.727	3.626	< 0.001
With	2	8,689.46	23.02	7	50,237.47	13.93	1.893	1.306	2.743	< 0.001
Season										
Spring	10	19,367.63	51.63	24	87,125.61	27.55	2.148	1.482	3.113	< 0.001
Summer	16	20,902.42	76.55	24	91,328.79	26.28	3.338	2.304	4.838	< 0.001
Autumn	10	26,263.26	38.08	25	102,001.30	24.51	1.780	1.229	2.580	< 0.001
Winter	17	21,153.10	80.37	28	87,626.93	31.95	2.883	1.989	4.177	< 0.001
Urbanization level										
1 (The highest)	24	31,046.48	77.30	35	106,638.51	32.82	2.699	1.863	3.912	< 0.001
2 The 2nd	21	40,157.87	52.29	41	168,804.41	24.29	2.468	1.703	3.576	< 0.001
3 The 3rd	5	4,768.95	104.84	15	28,516.79	52.60	2.284	1.576	3.310	< 0.001
4 (The lowest)	3	11,713.11	25.61	10	64,122.93	15.60	1.882	1.299	2.728	< 0.001
Level of care										
Hospital center	33	37,911.57	87.04	45	119,126.13	37.78	2.741	1.922	3.927	< 0.001
Regional hospital	16	36,999.23	43.24	31	165,211.64	18.76	2.641	1.823	3.828	< 0.001
Local hospital	4	12,775.61	31.31	25	83,744.88	29.85	1.202	0.830	1.742	0.279
PYs = Person-years; Rate: per 100,000 PYs; aHR = Adjusted Hazard ratio: Adjusted for the variables listed in Table 2.; CI = confidence interval										

Abbreviations: PKD = polycystic kidney disease, NT$ = new Taiwan dollar, DM = diabetes mellitus, HTN = hypertension, CAD = coronary artery disease, CHF = congestive heart failure, PAOD = peripheral arterial occlusive disease, Af = atrial fibrillation, COPD = chronic obstructive pulmonary disease, CKD = chronic kidney disease

**Table 4:** Factors of aortic dissection among patients with/without PKD in different CKD stages by using Cox regression

PKD	Population	Events	PYs	Rate	aHR	95% CI	95% CI	*P*
Without PKD	36,768	101	368,082.64	27.44	1.000			
Without CKD	33,637	91	338,065.87	26.92	1.125	0.784	1.629	0.215
With CKD	3,131	10	30,016.77	33.31	1.394	0.965	2.018	0.074
Without HD	1,425	4	14,152.48	28.26	1.181	0.813	1.714	0.187
With HD	1,706	6	15,864.29	37.82	1.580	1.099	2.296	0.001
With PKD	9,192	53	87,686.41	60.44	2.525	1.742	3.658	< 0.001
Without CKD	6,829	19	45,684.56	41.59	1.737	1.198	2.516	< 0.001
With CKD	2,363	34	42,001.85	80.95	3.382	2.334	4.896	< 0.001
Without HD	1,076	13	19,803.26	65.65	2.741	1.896	3.970	< 0.001
With HD	1,287	21	22,198.60	94.60	3.950	2.723	5.724	< 0.001

PYs = Person-years; Rate: per 100,000 PYs; aHR = Adjusted Hazard ratio: Adjusted for the variables listed in Table 2.; CI = confidence interval Abbreviations: PKD = polycystic kidney disease, CKD = chronic kidney disease, HD = hemodialysis
