# Supplementary material for: Impact of Chronic Kidney Disease on Aortic Dissection in Patients with Polycystic Kidney Disease: A Fifteen-year Nationwide Population-based Cohort Study in Taiwan
Source: Int J Med Sci. 2025 Feb 26;22(7):1493–503. doi: 10.7150/ijms.106518 (PMC11905265; doi:10.7150/ijms.106518)
Supplement: Supplementary file 1 — Supplementary figure. [file ijmsv22p1493s1.pdf]

Supplementary Figure

Figure S1. Joint effect for factors of AD stratified by PKD and HTN by using Cox regression

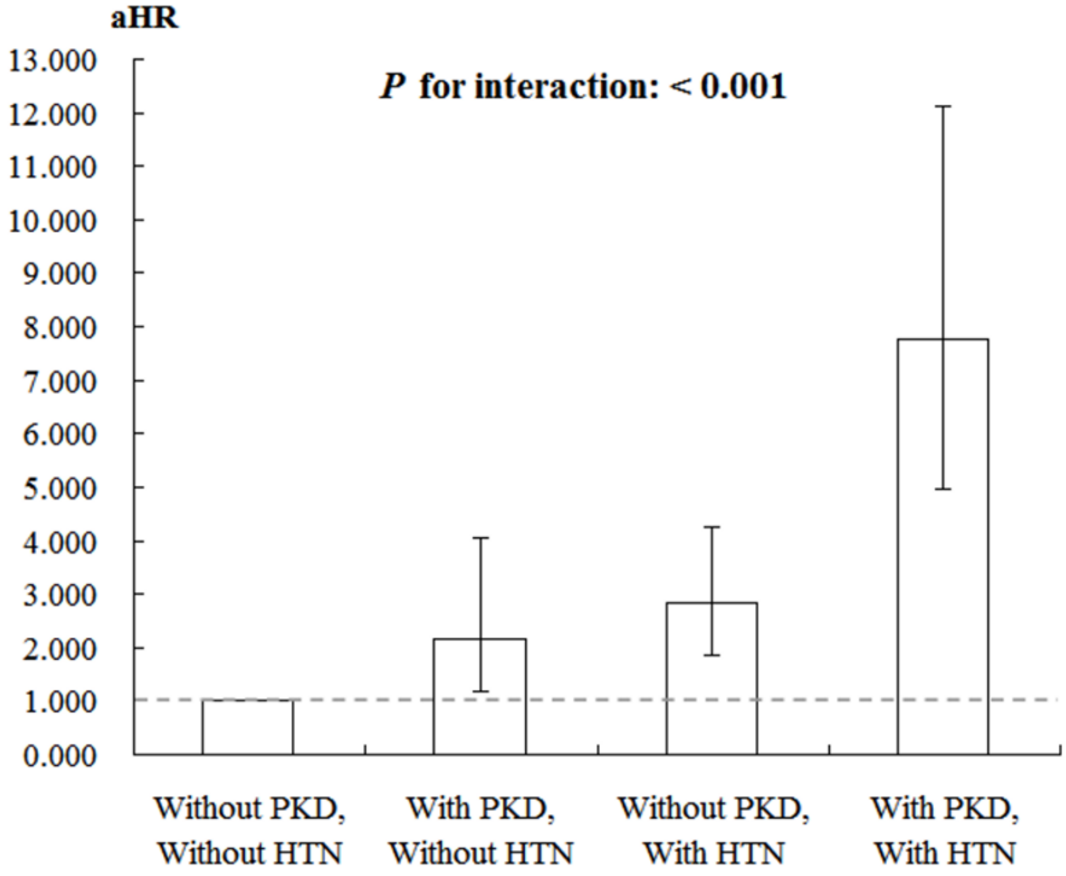

Abbreviations: AD = aortic dissection, HTN = hypertension, PKD = polycystic kidney disease
